# Oncogenic *Ras* cooperates with knockdown of the tumor suppressor *Lkb1* by RNAi to override organ size limits in Drosophila wing tissue

**DOI:** 10.17912/micropub.biology.000223

**Published:** 2020-03-03

**Authors:** Briana Brown Rackley, Evan Kiely, Chang-Soo Seong, Manali Rupji, Melissa Gilbert-Ross

**Affiliations:** 1 Department of Hematology and Medical Oncology, Emory University School of Medicine; 2 Biostatistics and Bioinformatics Shared Resource, Winship Cancer Institute of Emory University

**Figure 1:**
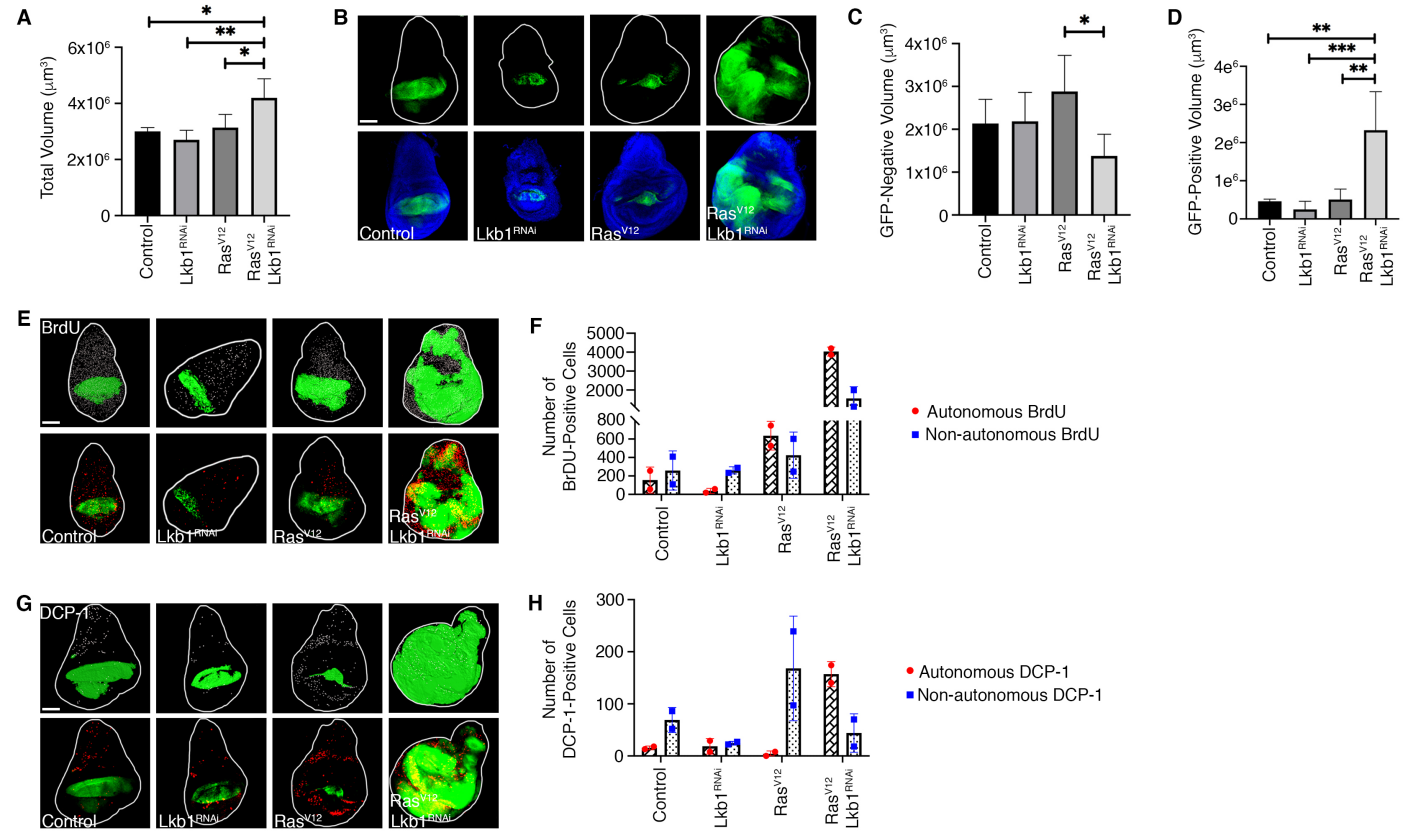
Co-expression of *Ras^V12^*/*Lkb1^RNAi^* in the *MS1096-Gal4* expression domain overrides organ size control. Quantification of 3^rd^ instar larval wing-imaginal disc total volume (A) and representative confocal images of wing imaginal discs expressing the indicated transgenes and GFP (green) in the *MS1096-Gal4* expression domain (B). DAPI (blue) labels cell nuclei. Quantification of GFP-negative volume (non-autonomous) (C), and GFP-positive volume (autonomous) (D) from the indicated genotypes. (E) Confocal images of 3^rd^ instar larval wing-imaginal discs carrying GFP-labeled wing pouch tissue labeled with BrdU. Top panel is a representative image of the IMARIS spot analysis used for quantification of BrdU positive cells. Bottom panel is actual immunofluorescence image of BrdU labeling (in red). Total number of autonomous and non-autonomous BrdU labeled cells are quantified in (F). (G) Confocal images of 3^rd^ instar larval wing-imaginal discs carrying GFP-labeled wing pouch tissue stained with an antibody to Death Caspase-1 (DCP-1). Top panel is a representative image of the IMARIS spot analysis used for quantification of DCP-1 positive cells. Bottom panel is actual immunofluorescence image of DCP-1 staining (in red). Total number of autonomous and non- autonomous DCP-1 stained cells are quantified in (H). Images are representative of 5-10 wing-imaginal discs per genotype. Scale bar, 100µm. Control = *MS1096-Gal4*,*w1118*. For graphs in (A), (C), and (D), bars represent mean volumes from 3-5 independent wing-imaginal discs per genotype and error bars represent standard deviation. Ordinary one-way ANOVA was conducted with significance assigned to *P* values<0.1. (A) p=0.0030 (C) p=0.0896 (D) p=0.0004. P-values between groups were compared with post-test. *p<0.1, **p<0.01, ***p<0.001. In (F) and (H) bars represent means from 2 independent wing-imaginal discs per genotype and error bars represent standard deviation. Significance was not analyzed due to sample size.

## Description

*KRAS* is the most frequently mutated oncogene in human cancer, particularly in cancers with a high mortality rate such as pancreatic, colorectal, and non-small cell lung cancer (NSCLC) (Ryan and Corcoran, 2018). While effective therapies directly targeting *KRAS*-mutant tumors have yet to be fully validated, recent clinical trials show positive progress for patients with the *KRAS(G12C)* mutation (Canon *et al.* 2019). Moreover, sequencing data has allowed for better understanding of how secondary mutations synergize with oncogenic *KRAS* to drive tumor progression. For example, activating mutations in *KRAS* frequently occur with loss-of-function mutations in the gene STK11, which encodes the tumor suppressor liver kinase B1 (*LKB1*), resulting in decreased patient survival, de novo resistance to targeted treatments and immunotherapies, and increased likelihood of tumor recurrence (Cancer Genome Atlas Research Network 2014, Skoulidis *et al.* 2018, Caiola *et al.* 2018). Additionally, previous work from genetically engineered mouse models (GEMMs) suggests loss of *Lkb1* is sufficient to promote the progression and metastasis of nascent *Kras*-driven lung adenocarcinoma (Ji *et al.* 2007). Therefore, we sought to determine whether knockdown of *Lkb1* by RNAi could cooperate with activating mutations in *Ras* to drive tissue overgrowth in wing imaginal discs of the genetically tractable model organism *Drosophila melanogaster*.

To address this question, we obtained transgenic *Drosophila* expressing oncogenic *Ras^V12^*, which on its own causes hyperplastic growth balanced by non-autonomous cell death in imaginal tissues (Karim and Rubin 1998). To knockdown *Lkb1* we obtained an RNAi fly stock (*Lkb1^RNAi^*) developed by the Transgenic RNAi Project (TRiP) (Dietzl *et al.* 2007) and validated through the Harvard Medical School RNAi Stock Validation and Phenotypes (RSVP) resource (Perkins *et al.* 2015). Of note, the *Lkb1^RNAi^* stock was determined to have approximately 68% knockdown efficiency when used with the *MTD-Gal4* driver (Sopko *et al.* 2014). Additional validation using the Updated Targets of RNAi Reagents (UP-TORR) Fly resource confirmed no off-target effects with this RNAi sequence (Hu *et al.* 2013). We generated a combined *Ras^V12^/Lkb1^RNAi^*fly line, and crossed our double mutant (along with single transgenes as controls) to the *MS1096-Gal4, UAS-GFP* wing pouch driver. In order to precisely measure effects on overall organ size, we used confocal microscopy to acquire *z*-sections through the entire wing disc, followed by 3D reconstruction and volume measurements using IMARIS software. We determined that total wing disc volume was significantly larger in *MS1096-Gal4*; *Ras^V12^*/*Lkb1^RNAi^*tissues compared to control genotypes. (A-B). Previous investigations have shown that *Lkb1* can exert a non-autonomous role in tumor suppression (Katajisto *et al.* 2008; Tanwar *et al.* 2012; Ollila *et al.* 2018). Therefore, we investigated whether the increase in organ size was due to autonomous vs. non-autonomous effects on growth. To do this we measured individual volumes of GFP-positive and GFP-negative tissue across genotypes. Expression of *Ras^V12^*/*Lkb1*^RNAi^ led to significant autonomous overgrowth in the GFP-positive *MS1096* expression domain, while the GFP-negative (non-autonomous) tissue compartment remained unchanged (C-D).

Changes in organ size control can result from any number of combinations of cell growth, proliferation, and cell death phenotypes. To investigate the compartmental effects on cell proliferation and cell death in *Ras^V12^*/*Lkb1*^RNAi^ tissues, we used the *MS1096-Gal4* driver to express *Lkb1^RNAi^*, *Ras^V12^*, or *Ras^V12^*/*Lkb1^RNAi^* in developing wing pouches. Tissues were either labeled with BrdU or an anti-Death Caspase-1 (DCP-1) antibody (E-F, G-H). Knockdown of *Lkb1* alone resulted in no change in the absolute levels of BrdU or DCP-1 relative to control discs (F, H). Expression of *Ras^V12^* alone resulted in a mild increase in the amount of autonomous BrdU and non-autonomous DCP-1 (F, H). Alternatively, co-expression of *Ras^V12^*/*Lkb1^RNAi ^*led to a dramatic shift in cellular phenotypes with a large increase in autonomous and non- autonomous BrdU – and a rescue of the non-autonomous cell death observed in cells expressing *Ras^V12^*alone. Therefore, knockdown of *Lkb1* in the context of oncogenic *Ras* in the *Drosophila* wing pouch can exert both non-autonomous and autonomous effects that override organ size control. Future studies will focus on the signaling pathways responsible for both phenotypes which could represent novel, targetable pathways for the thousands of cancer patients in the U.S. with *LKB1* mutations.

## Methods

**Immunostaining and Confocal Microscopy.** 3^rd^ instar larval wing-imaginal discs were dissected in 1X phosphate- buffered saline (PBS) and fixed in 4% paraformaldehyde for 30 minutes on ice. Discs were then washed three times for 10 minutes each in ice cold 1X PBS, permeabilized in 0.3% Triton X100/1X PBS (PBST) for 20 minutes at RT, and washed again three times for 10 minutes each before blocking in 10% normal goat serum in 0.1% PBST for 30 minutes at RT. Discs were incubated in primary antibodies (4°C overnight) in 10% normal goat serum (NGS)/0.1% PBST. The following day, discs were washed three times for five minutes each in 0.1% PBST before incubating in secondary antibodies (in the dark at RT for one hour) in 10% NGS/0.1% PBST. Finally, discs were washed three times for 10 minutes each in 1X PBS at RT and mounted using VectaShield anti-fade mounting medium. Fluorescent secondary antibody was from Jackson ImmunoResearch. Fluorescent images were taken on a Leica MZ10F (× 1 0.08899 NA) or Leica TCS SP8 inverted confocal microscope (× 10 air HC PL Fluotar, 0.3 NA, × 20 air HC PL APO, 0.75 NA, or × 40 oil HC PL APO, 1.30 NA) using 0.5 μm z-stack intervals and sequential scanning (405 nm DMOD Flexible, 488 nm argon, 514 nm argon).

**BrdU Labeling.** 3^rd^ instar larval wing-imaginal discs were dissected in Grace’s Insect Medium (ThermoFisher) then transferred into Grace’s Insect Medium containing 0.25mg/ml BrdU (Invitrogen B23151) and incubated at 25°C for 90 minutes. Discs were then washed in Grace’s Insect Medium for five minutes on ice followed by washing two times for five minutes each in 1X PBS on ice. Discs were fixed overnight (wrapped in foil) in 1% paraformaldehyde/0.05%

Tween 20. The following day discs were washed three times for five minutes each in 1X PBS and permeabilized for 20 minutes at RT in 0.3% PBST. To remove detergent, discs were washed five times for five minutes each in 1X PBS and DNAse treated for 30 minutes at 37°C. Discs were then washed three times for 10 minutes each in 0.1% PBST and incubated overnight at 4°C in primary antibody. The next day, discs were washed 5 times for a total of 30 minutes with 0.1% PBST and incubated overnight in secondary antibody from Cell Signaling. Finally, discs were washed three times for 10 minutes each in 0.1% PBST and mounted in VectaShield anti-fade mounting medium.

**Image Processing and Quantification.** IMARIS microscopy image analysis software was used for all image processing and quantification. After file conversion into IMARIS, minimum and maximum intensity values were established for each channel and maintained across genotypes. Based on these values, regions of interest, termed “masks”, were created for DAPI and GFP channels. These masks were used to determine total volume, GFP-positive volume, and GFP-negative volume. The “Spot” feature was used to correctly identify cells labeled with BrdU or stained with DCP-1. Spot size was constrained to 2.5 and spot quality was restricted to greater than 7.07 for all genotypes. Autonomous BrdU incorporation and DCP-1 staining was calculated by taking the number of “spots” occurring within the GFP-marked “mask”. Values were then graphed and statistically analyzed using Prism GraphPad 8. Ordinary one-way ANOVA with a Tukey’s multiple comparisons test was conducted with significance assigned to *P* values ≤ 0.1.

## Reagents

The following *Drosophila* stocks were used:

*P{UAS-Ras85D.V12} (UAS-Ras^V12^)* – BDSC 64196

y[1] sc[*] v[1]; P{y[+t7.7] v[+t1.8]= TRiP.HMS01351}attP2 (*UAS*–*Lkb1^RNAi^*) – BDSC 34362

*w^1118^*– BDSC 3605 (gift from K. Moberg – Emory University)

*MS1096-Gal4, UAS-GFP* (Derived fromBDSC 8860)(gift from K. Moberg – Emory University)

The following antibodies were used:

rabbit anti-cleaved Drosophila DCP-1 (Asp216) (Cell Signaling, 1:100)

mouse anti-BrdU primary antibody (B44) (BD, 1:50)

goat anti-rabbit Cy3 AffiniPure secondary antibody (Jackson ImmunoResearch 1:400)

goat anti-mouse F(ab)’2 AlexaFluor-555 secondary antibody (Cell Signaling, 1:500)
